# Using the sample maximum to estimate the parameters of the underlying distribution

**DOI:** 10.1371/journal.pone.0215529

**Published:** 2019-04-25

**Authors:** Alex Capaldi, Tiffany N. Kolba

**Affiliations:** Department of Mathematics and Statistics, Valparaiso University, Valparaiso, IN, United States of America; Dartmouth College Geisel School of Medicine, UNITED STATES

## Abstract

We propose novel estimators for the parameters of an exponential distribution and a normal distribution when the only known information is a sample of sample maxima; i.e., the known information consists of a sample of *m* values, each of which is the maximum of a sample of *n* independent random variables drawn from the underlying exponential or normal distribution. We analyze the accuracy and precision of the estimators using extreme value theory, as well as through simulations of the sampling distributions. For the exponential distribution, the estimator of the mean is unbiased and its variance decreases as either *m* or *n* increases. Likewise, for the normal distribution, we show that the estimator of the mean has negligible bias and the estimator of the variance is unbiased. While the variance of the estimators for the normal distribution decreases as *m*, the number of sample maxima, increases, the variance increases as *n*, the sample size over which the maximum is computed, increases. We apply our method to estimate the mean length of pollen tubes in the flowering plant *Arabidopsis thaliana*, where the known biological information fits our context of a sample of sample maxima.

## 1 Introduction

Consider the scenario where one has obtained data where each observation is the maximum value of *n* independent, identically distributed random variables drawn from either an exponential distribution or a normal distribution with unknown parameters. That is, $X_{ij}\overset{\text{iid}}{∼}Exp\left(\beta \right)$ or $X_{ij}\overset{\text{iid}}{∼}N\left(\mu ,\sigma^{2}\right)$ for *i* = 1, …, *n* and known data is drawn from $Y_{j}=\text{max}{\left\{X_{ij}\right\}}_{i=1}^{n}$ for *j* = 1, …, *m*. Here we present a process to estimate the mean *β* of the underlying exponential distribution or the mean *μ* and variance *σ*^2^ of the underlying normal distribution from only the set of *Y*_*j*_’s.

Much previous research has been conducted in the field of extreme value theory on the distribution of the maximum from a sample of *n* independent, identically distributed random variables. In particular, the Fisher-Tippett-Gnedenko theorem states that the distribution of the sample maximum, after proper rescaling, can only converge to one of three types of distributions: the Gumbel distribution, the Fréchet distribution, or the Weibull distribution [1]. When the original underlying distribution is exponential or normal, the limiting distribution of the rescaled sample maximum is the Gumbel distribution. Extreme value theory has been applied in many applications, such as estimating the probability of an extreme flood, severe adverse side effect of a drug, maximum environmental load on a structure, or large insurance loss [2]. In these applications, the underlying distribution and its parameters are typically known and the focus is on estimating the probability distribution of the sample maximum. The focus of our scenario is novel in that we are using a known sample of sample maxima to estimate unknown parameters of the underlying distribution.

Standard techniques for estimating unknown parameters, such as method of moments or maximum likelihood estimation, typically assume that the known information consists of a sample of observations drawn directly from the underlying population distribution. However, in our scenario under consideration, the direct observations are unknown. Rather, we only know the maximum value of each sample of direct observations. The estimators we propose in the following sections are the first, to our knowledge, for estimating unknown population parameters when the only known information is a sample of sample maxima.

## 2 Estimator for exponential distribution

We begin by considering the case where the underlying distribution is exponential with unknown mean *β*. In Theorem 1 below, we propose an estimator for *β* and compute its expected value and variance.

**Theorem 1**. *Let* $X_{ij}\overset{iid}{∼}Exp\left(\beta \right)$ *for i* = 1, …, *n and* $Y_{j}=\text{max}{\left\{X_{ij}\right\}}_{i=1}^{n}$ *for j* = 1, …, *m*. *Set*

$$\hat{\beta }=\frac{\bar{Y}}{H_{n}}=\frac{1}{mH_{n}}\sum_{j=1}^{m}Y_{j}.$$ (1)

*Then*

$$E\left(\hat{\beta }\right)=\beta ,\;var\left(\hat{\beta }\right)=\frac{\beta^{2}G_{n}}{mH_{n}^{2}}$$ (2)

*where*

$$H_{n}=\sum_{i=1}^{n}\frac{1}{i},\;G_{n}=\sum_{i=1}^{n}\frac{1}{i^{2}}.$$

*Proof*. From the formula for $\hat{\beta }$ given in Eq (1), it directly follows that

$$E\left(\hat{\beta }\right)=\frac{E(Y_{j})}{H_{n}},\;\text{var}\left(\hat{\beta }\right)=\frac{\text{var}(Y_{j})}{mH_{n}^{2}}.$$

Hence, it only remains to compute the expected value and variance of the maximum of a single sample of *n* independent *Exp*(*β*) random variables. Let *X*_(*i*)_ denote the *i*th smallest observation from such a sample. Then *Y*_*j*_ = *X*_(*n*)_ can be decomposed as the following telescoping sum:

$$Y_{j}=X_{\left(n\right)}=X_{\left(1\right)}+\left(X_{\left(2\right)}-X_{\left(1\right)}\right)+\cdots +\left(X_{\left(n\right)}-X_{\left(n-1\right)}\right).$$

Due to the memoryless property of the exponential distribution, *X*_(2)_ − *X*_(1)_ is independent of *X*_(1)_. Moreover, while *X*_(1)_ is the minimum of *n* independent *Exp*(*β*) random variables, *X*_(2)_ − *X*_(1)_ can be viewed as the minimum of a sample of *n* − 1 independent *Exp*(*β*) random variables. Likewise, all of the terms in the telescoping sum for *Y*_*j*_ = *X*_(*n*)_ are independent with *X*_(*i*+1)_ − *X*_(*i*)_ equal in distribution to the minimum of a sample of *n* − *i* independent *Exp*(*β*) random variables, which in turn is equal in distribution to *Exp*(*β*/(*n* − *i*)). Thus,

$$E\left(Y_{j}\right)=E\left(X_{\left(n\right)}\right)=\frac{\beta }{n}+\frac{\beta }{n-1}+\cdots +\frac{\beta }{2}+\beta =\beta \sum_{i=1}^{n}\frac{1}{i}=\beta H_{n}$$

and

$$\text{var}\left(Y_{j}\right)=\text{var}\left(X_{\left(n\right)}\right)=\frac{\beta^{2}}{n^{2}}+\frac{\beta^{2}}{{\left(n-1\right)}^{2}}+\cdots +\frac{\beta^{2}}{2^{2}}+\beta^{2}=\beta^{2}\sum_{i=1}^{n}\frac{1}{i^{2}}=\beta^{2}G_{n}.$$

Substituting these expressions for *E*(*Y*_*j*_) and var(*Y*_*j*_) into the equations for the expected value and variance of $\hat{\beta }$ produces the formulas given in Eq (2).

As a consequence of Theorem 1, we have shown that $\hat{\beta }$ is an unbiased estimator for *β* and that its variance decreases at a rate proportional to $\frac{1}{m}$. Since $G_{n}\to \frac{\pi^{2}}{6}$ and *H*_*n*_ → ∞ at rate log *n* as *n* → ∞, the variance of $\hat{\beta }$ decreases at a rate proportional to $\frac{1}{{\left(\text{log}\;n\right)}^{2}}$. Thus, the precision of the estimator $\hat{\beta }$ can be improved more rapidly by increasing *m*, the number of sample maxima, compared to increasing *n*, the sample size over which the maximum is computed.

## 3 Estimators for normal distribution

We now consider the case where the underlying distribution is normal with unknown mean *μ* and unknown variance *σ*^2^. In Theorem 2 below, we propose estimators for *μ* and *σ*^2^ and analyze their expected value, while in Theorem 3 we analyze the variance of the estimators.

**Theorem 2**. *Let* $X_{ij}\overset{iid}{∼}N\left(\mu ,\sigma^{2}\right)$ *for i* = 1, …, *n and* $Y_{j}=\text{max}{\left\{X_{ij}\right\}}_{i=1}^{n}$ *for j* = 1, …, *m*. *Let* $\bar{Y}$ *and* $S_{Y}^{2}$ *denote the sample mean and sample variance, respectively, of the Y*_*j*_’*s*. *Set*

$$\hat{\mu }=\bar{Y}-\frac{k_{n}}{\sqrt{c_{n}}}S_{Y},\;{\hat{\sigma }}^{2}=\frac{S_{Y}^{2}}{c_{n}}$$ (3)

*where k*_*n*_ *denotes the mean and c*_*n*_ *denotes the variance of the maximum of n independent, identically distributed N*(0, 1) *random variables*. *Then* $E\left({\hat{\sigma }}^{2}\right)=\sigma^{2}$, *while* $E(\hat{\mu })>\mu$ *with* $E(\hat{\mu })\to \mu$ *as m* → ∞.

*Proof*. The cumulative distribution function of *Y*_*j*_ is given by

$$\begin{array}{cc} F_{Y_{j}}\left(y\right) & =P\left(Y_{j}\leq y\right)={\left[P\left(X_{ij}\leq y\right)\right]}^{n}\\ & ={\left[P(\frac{X_{ij}-\mu }{\sigma }\leq \frac{y-\mu }{\sigma })\right]}^{n}={\left[\Phi (\frac{y-\mu }{\sigma })\right]}^{n}. \end{array}$$

Differentiating, the probability density function of *Y*_*j*_ is

$$f_{Y_{j}}\left(y\right)=n{\left[\Phi (\frac{y-\mu }{\sigma })\right]}^{n-1}\phi (\frac{y-\mu }{\sigma })\frac{1}{\sigma },$$

where Φ(*z*) denotes the cumulative distribution function and *ϕ*(*z*) denotes the probability density function of a *N*(0, 1) random variable. The expected value of *Y*_*j*_ can then be calculated as

$$\begin{array}{cc} E(Y_{j}) & =\int_{-\infty }^{\infty }yf_{Y_{j}}\left(y\right)dy=\int_{-\infty }^{\infty }yn{\left[\Phi (\frac{y-\mu }{\sigma })\right]}^{n-1}\phi (\frac{y-\mu }{\sigma })\frac{1}{\sigma }dy\\ & =\mu +\sigma \int_{-\infty }^{\infty }zn{\left[\Phi (z)\right]}^{n-1}\phi (z)dz=\mu +\sigma k_{n}. \end{array}$$

We also obtain that the variance of *Y*_*j*_ is

$$\begin{array}{cc} \text{var}(Y_{j}) & =\int_{-\infty }^{\infty }{\left(y-E\left(Y_{j}\right)\right)}^{2}f_{Y_{j}}\left(y\right)dy\\ & =\int_{-\infty }^{\infty }{\left(y-\mu -\sigma k_{n}\right)}^{2}n{\left[\Phi (\frac{y-\mu }{\sigma })\right]}^{n-1}\phi (\frac{y-\mu }{\sigma })\frac{1}{\sigma }dy\\ & =\sigma^{2}\int_{-\infty }^{\infty }{\left(z-k_{n}\right)}^{2}n{\left[\Phi (z)\right]}^{n-1}\phi (z)dz=\sigma^{2}c_{n}. \end{array}$$

We can now use the equations for *E*(*Y*_*j*_) and var(*Y*_*j*_) to compute the expected value of the estimators. In particular, the expected value of the estimator of the variance is

$$E\left({\hat{\sigma }}^{2}\right)=\frac{E(S_{Y}^{2})}{c_{n}}=\frac{\text{var}(Y_{j})}{c_{n}}=\frac{\sigma^{2}c_{n}}{c_{n}}=\sigma^{2},$$

while the estimator of the mean is

$$E\left(\hat{\mu }\right)=E\left(Y_{j}\right)-\frac{k_{n}}{\sqrt{c_{n}}}E\left(S_{Y}\right)>\left(\mu +\sigma k_{n}\right)-\frac{k_{n}}{\sqrt{c_{n}}}\left(\sigma \sqrt{c_{n}}\right)=\mu .$$

Although Jensen’s inequality implies that $\hat{\mu }$ has positive bias since $E\left(S_{Y}\right)<\sqrt{E(S_{Y}^{2})}=\sqrt{\text{var}(Y_{j})}$, the bias of the sample standard deviation goes to zero as the sample size increases. Hence, $E(\hat{\mu })\to \mu$ as *m* → ∞.

Note that the constants *k*_*n*_ and *c*_*n*_ that appear in the formulas for the estimators depend upon only the sample size *n*. Exact integral expressions exist for *k*_*n*_ and *c*_*n*_ and are given in the proof of Theorem 2, but the integrals cannot be evaluated in closed form. However, the constants can be approximated either analytically or numerically.

In [3], Cramér showed that *b*_*n*_(*Z*_(*n*)_ − *b*_*n*_) converges in distribution to the standard Gumbel distribution, where *Z*_(*n*)_ is the maximum of a sample of *n* independent, identically distributed *N*(0, 1) random variables and

$$b_{n}={\left(2\text{log}n\right)}^{\frac{1}{2}}-\frac{\frac{1}{2}\text{log}\left(4\pi \text{log}n\right)}{{\left(2\text{log}n\right)}^{\frac{1}{2}}}.$$

Since the standard Gumbel distribution has mean equal to *γ* ≈ 0.5772, the Euler-Mascheroni constant, and variance equal to $\frac{\pi^{2}}{6}$, we can use these values to approximate

$$k_{n}\approx \frac{\gamma }{b_{n}}+b_{n},\;c_{n}\approx \frac{\pi^{2}}{6b_{n}^{2}}.$$ (4)

**Fig 1:**
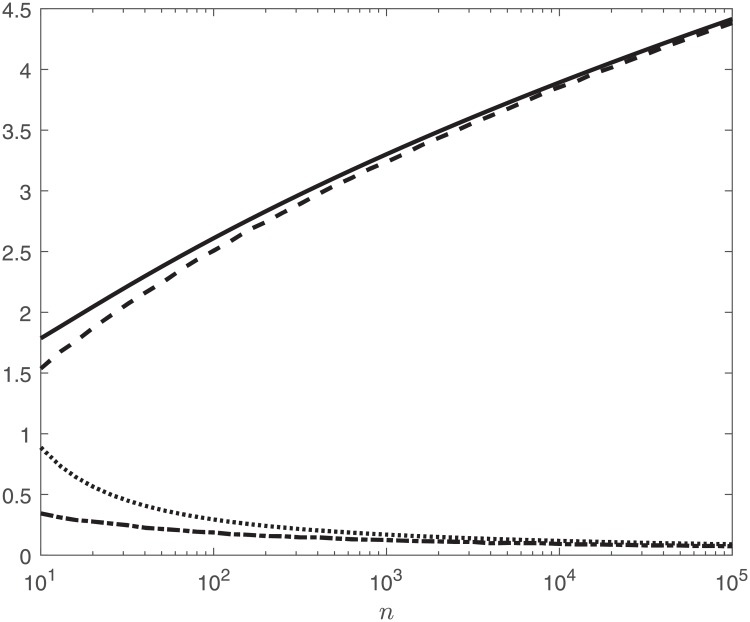
From the top curve to the bottom, the plot displays the values of the analytic approximation for *k*_*n*_ (solid) and the numerical approximation for *k*_*n*_ (dashed), along with the analytic approximation for *c*_*n*_ (dotted) and the numerical approximation for *c*_*n*_ (dot-dashed).

Fig 1 displays the values of the analytic approximations for *k*_*n*_ and *c*_*n*_ given in Eq (4) for *n* ranging from 10 to 100,000, along with corresponding numerical approximations, plotted on a semi-log scale. The numerical approximations for *k*_*n*_ and *c*_*n*_ were computed from 10,000 realizations of a simulation of the maximum.

**Fig 2:**
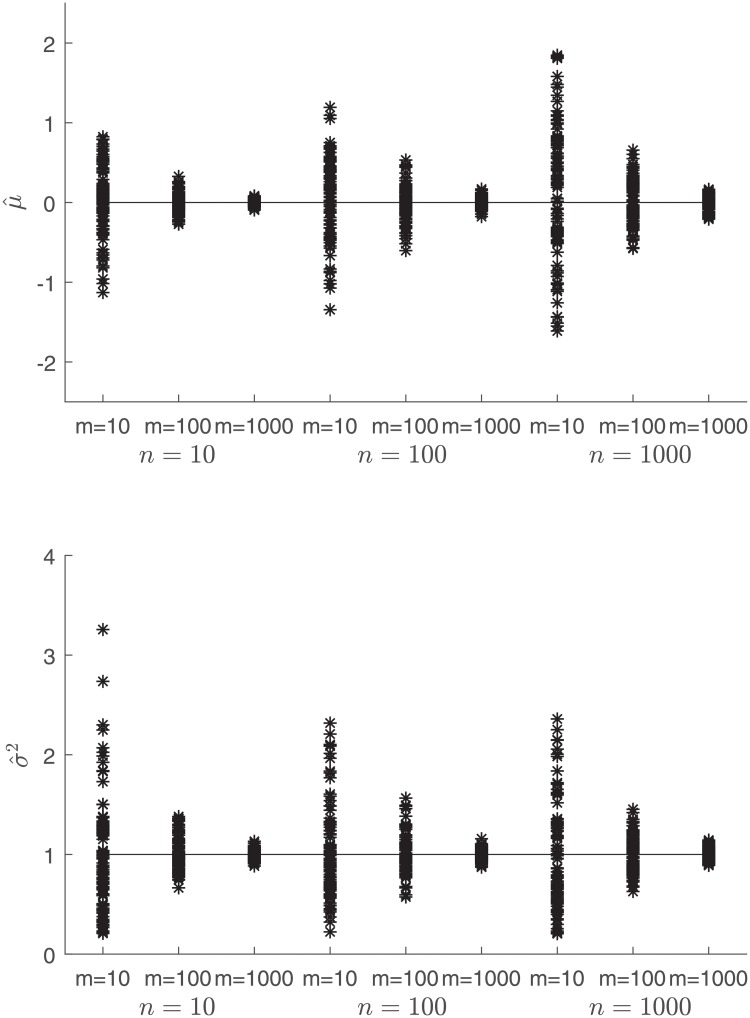
Estimates of $\hat{\mu }$ (top) and ${\hat{\sigma }}^{2}$ (bottom) from 100 realizations with *n*, *m* = 10, 100, and 1000. The horizontal lines indicate the true values of *μ* and *σ*^2^.

While Theorem 2 showed that $\hat{\mu }$ is positively biased with the bias approaching zero as *m* → ∞, the estimation bias is fairly minimal even for relatively small values of *m*. Fig 2 displays the sampling distributions of the estimators for *μ* and *σ*^2^ for varying values of *m* and *n*. In all the simulations, the sampling distribution of $\hat{\mu }$ is fairly centered around the true value of *μ* = 0. Setting the true value of *μ* to a nonzero value simply shifts the sampling distribution of $\hat{\mu }$ and has no effect on ${\hat{\sigma }}^{2}$. We also observe from Fig 2 that the variability of the sampling distributions of the estimators decreases as *m*, the number of sample maxima, increases, but increases as *n*, the sample size over which the maximum is computed, increases. We derive an analytical justification for this behavior in Theorem 3 below.

**Theorem 3**. *Let* $X_{ij}\overset{iid}{∼}N\left(\mu ,\sigma^{2}\right)$ *for i* = 1, …, *n and* $Y_{j}=\text{max}{\left\{X_{ij}\right\}}_{i=1}^{n}$ *for j* = 1, …, *m*. *Let* $\bar{Y}$ *and* $S_{Y}^{2}$ *denote the sample mean and sample variance, respectively, of the Y*_*j*_’*s*. *Set*

$$\hat{\mu }=\bar{Y}-\frac{k_{n}}{\sqrt{c_{n}}}S_{Y},\;{\hat{\sigma }}^{2}=\frac{S_{Y}^{2}}{c_{n}}$$

*where k*_*n*_ *denotes the mean and c*_*n*_ *denotes the variance of the maximum of n independent, identically distributed N*(0, 1) *random variables*. *Then*

$$var\left({\hat{\sigma }}^{2}\right)=\sigma^{4}(\frac{2}{m-1}+\frac{\kappa }{m})$$

*and*

$$var\left(\hat{\mu }\right)\approx \frac{\sigma^{2}c_{n}}{m}+\frac{\sigma^{2}k_{n}^{2}}{4}(\frac{2}{m-1}+\frac{\kappa }{m})-\frac{\sigma^{2}k_{n}\sqrt{c_{n}}\gamma_{1}}{m}$$

*where γ*_1_ *is the skewness and κ is the excess kurtosis of the distribution of Y*_*j*_.

*Proof*. The variance of $S_{Y}^{2}$ can be computed as

$$\text{var}\left(S_{Y}^{2}\right)={\left(\text{var}\left(Y_{j}\right)\right)}^{2}(\frac{2}{m-1}+\frac{\kappa }{m}),$$

where *κ* is the excess kurtosis of the distribution of *Y*_*j*_ [4]. Since we computed that var(*Y*_*j*_) = *σ*^2^*c*_*n*_ in Theorem 2, we obtain the desired result

$$\text{var}\left({\hat{\sigma }}^{2}\right)=\text{var}(\frac{S_{Y}^{2}}{c_{n}})=\sigma^{4}(\frac{2}{m-1}+\frac{\kappa }{m})$$

for the variance of the variance estimator. Now for the variance of the mean estimator, we compute that

$$\text{var}\left(\hat{\mu }\right)=\text{var}(\bar{Y}-\frac{k_{n}}{\sqrt{c_{n}}}S_{Y})=\text{var}\left(\bar{Y}\right)+\frac{k_{n}^{2}}{c_{n}}\text{var}\left(S_{Y}\right)-2\frac{k_{n}}{\sqrt{c_{n}}}\text{cov}\left(\bar{Y},S_{Y}\right).$$

To simplify the above expression, we use the fact that $\text{var}\left(\bar{Y}\right)=\frac{\text{var}(Y_{j})}{m}$, along with the approximations

$$\text{var}\left(S_{Y}\right)\approx \frac{\text{var}(S_{Y}^{2})}{4\text{var}(Y_{j})},\;\text{cov}\left(\bar{Y},S_{Y}\right)\approx \frac{\text{cov}(\bar{Y},S_{Y}^{2})}{2\sqrt{\text{var}(Y_{j})}}=\frac{\text{var}\left(Y_{j}\right)\gamma_{1}}{2m},$$

where *γ*_1_ is the skewness of the distribution of *Y*_*j*_ [5]. Using these approximations, we obtain the desired result

$$\text{var}\left(\hat{\mu }\right)\approx \frac{\sigma^{2}c_{n}}{m}+\frac{\sigma^{2}k_{n}^{2}}{4}(\frac{2}{m-1}+\frac{\kappa }{m})-\frac{\sigma^{2}k_{n}\sqrt{c_{n}}\gamma_{1}}{m}$$

for the variance of the mean estimator.

As *n* increases, the value of *κ* increases monotonically from 0, the excess kurtosis of the normal distribution, to 12/5, the excess kurtosis of the Gumbel distribution [1]. Thus, from Theorem 3, we observe that the variance of the sampling distribution of ${\hat{\sigma }}^{2}$ increases proportionally to *σ*^4^ as *σ* increases and decreases proportionally to 1/*m* as *m* increases, but only slightly increases as *n* increases.

As with the excess kurtosis, the skewness of the distribution of *Y*_*j*_ increases monotonically from the value for the normal distribution, i.e., *γ*_1_ = 0, to the value for the standard Gumbel distribution, i.e., *γ*_1_ ≈ 1.13955, as *n* increases [6]. Since the constant *c*_*n*_ decreases towards 0 while the constant *k*_*n*_ increases towards infinity, the dominant term in the variance of $\hat{\mu }$ increases proportionally to $k_{n}^{2}$ as *n* increases. We also observe from Theorem 3 that the variance of the sampling distribution of $\hat{\mu }$ increases proportionally to *σ*^2^ as *σ* increases and decreases proportionally to 1/*m* as *m* increases. These relationships explain the behavior of the sampling distributions displayed in Fig 2.

## 4 Biological application

During fertilization in flowering plants, once pollen land on the stigma, the pollen will grow tubes that travel down through a transmitting tract from the stigma toward an ovule. Pollen compete against each other in a race towards the limited number of ovules to determine which pollen will father the seeds. The mean length of the population of pollen tubes at various time points is of interest to plant biologists, yet, to date, there are only measures of the lengths of the *longest* pollen tubes in such competitions [7]. Since the pollen tube lengths must have a positive value, it is reasonable to assume that the lengths follow an exponential distribution. Hence, our method described in Section 2 will allow the mean pollen tube length to be estimated given the structure of the experimental data.

In [7], Swanson et al. measured the longest pollen tube lengths at four time points for two accessions (i.e., specific geographical populations) of *Arabidopsis thaliana* in a laboratory setting. For both the Columbia and Landsberg accessions, either *m* = 8 or *m* = 9 individual plants were used for each time point. The average number of pollen tubes within each plant was *n* = 933 for the Columbia accession and *n* = 727 for the Landsberg accession. Table 1 reports the sample mean of the longest pollen tube from the *m* plants for each accession after 3, 6, 9, and 24 hours. Using these sample means of the longest lengths and Eq (2) from Theorem 1, we then estimated the overall mean length for each accession at each time point. The resulting estimates and their standard errors are listed in Table 1.

**Table 1:** The sample mean, $\bar{y}$, of the longest pollen tube from *m* plants, where each plant contained an average of *n* pollen tubes, along with the corresponding estimated mean pollen length, $\hat{\beta }$, and its standard error, for two accessions of *Arabidopsis thaliana* at varying time points.

Accession	time (hrs)	*m*	$\bar{y}$ (mm)	$\hat{\beta }$ (mm)	${\text{SE}}_{\hat{\beta }}$ (mm)
Columbia (*n* = 933)	3	9	0.690	0.093	0.005
	6	8	1.069	0.144	0.009
	9	9	2.538	0.342	0.020
	24	9	2.778	0.375	0.022
Landsberg (*n* = 727)	3	9	0.474	0.066	0.004
	6	8	0.676	0.094	0.006
	9	8	1.795	0.251	0.016
	24	9	2.325	0.324	0.019

**Fig 3:**
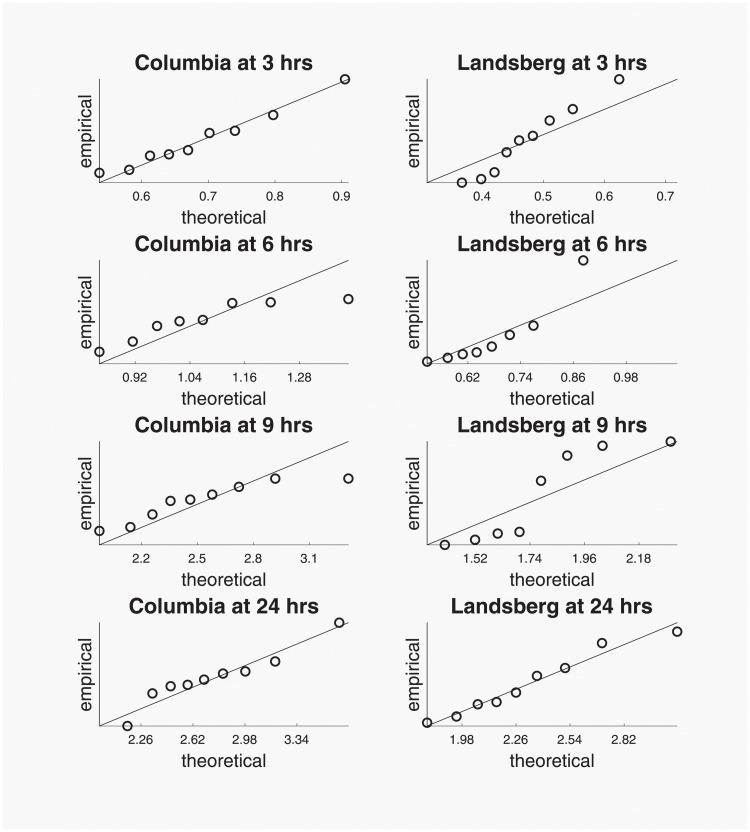
Q-Q plots of the distribution of the maximum pollen tube length from laboratory experiments versus the distribution of the maximum from an exponential distribution.

To further evaluate the validity of the assumption that the population of pollen tube lengths is exponentially distributed, we produced Q-Q plots of the distribution of the maximum pollen tube length from the laboratory experiments performed by Swanson et al. versus the distribution of the maximum from an exponential distribution. The theoretical distribution of the maximum from an exponential distribution was simulated using 10,000 realizations of $Y_{j}=\text{max}{\left\{X_{ij}\right\}}_{i=1}^{n}$ where $X_{ij}\overset{\text{iid}}{∼}Exp\left(\hat{\beta }\right)$ for *i* = 1, …, *n*, using the values of *n* and $\hat{\beta }$ that are listed in Table 1. The Q-Q plots, displayed in Fig 3, show a roughly linear relationship, supporting the assumption that the underlying distribution of pollen tube lengths is exponential. Moreover, we performed a Kolmogorov-Smirnov test of the equality of the empirical and theoretic distributions for each accession of *Arabidopsis thaliana* at each time point. The smallest resulting p-value was 0.52 (corresponding to the Landsberg accession at 9 hours), further indicating that there is no evidence that the distribution of pollen tube lengths differs significantly from an exponential distribution.
